# Plan-do-check-act (PDCA) cycle analysis for antimicrobial stewardship of orthopedic patients in trauma center—implementation research

**DOI:** 10.1017/ash.2026.10374

**Published:** 2026-05-11

**Authors:** Rishin Raj P, Nusrat Shafiq, Niveditha K, Deepa Kumari, Mandeep Singh Dhillon, Pankaj Arora, Devendra Kumar Chouhan, Uttam Chand Saini, Ankit Dadra, Archana Angrup, Manisha Biswal, Shivam Maheshwari, Aditya Gupta, Manjunath Nishani, Rahul Yadav, Udit Jayant, Kamal Singh Ladwal, Supreeth K

**Affiliations:** 1 Department of Pharmacology, Post Graduate Institute of Medical Education and Research, Chandigarh 160012, India; 2 Clinical Pharmacology Unit, Post Graduate Institute of Medical Education and Research, Chandigarh 160012, India; 3 Department of Orthopaedics, Post Graduate Institute of Medical Education and Research, Chandigarh 160012, India; 4 Department of Hospital Administration, Chandigarh 160012, India; 5 Department of Microbiology, Post Graduate Institute of Medical Education and Research, Chandigarh 160012, India

## Abstract

**Objective:**

The current study was done to evaluate the impact of a plan-do-check-act (PDCA)-based AMS program implemented at a tertiary trauma center.

**Setting/Patients:**

Inpatients admitted to an Advanced Trauma Centre of a tertiary care hospital in North India.

**Methods:**

This implementation research study was conducted in four phases from March 2019 to November 2024. The AMS strategies included prospective audits, feedback mechanisms, education, and multidisciplinary collaboration. Descriptive statistics were used for antibiotic prescription practices, adherence to guidelines, and patient outcomes. Metrics for antibiotics utilization and appropriateness of prescription were the key performance indicators analyzed.

**Results:**

A total of 767 patients were enrolled during this period. The practice of antibiotic prescription changed from being prophylactic to culture-based—increasing from 0.7% in phase 1 to 21.4% in phase 4. Compliance to feedback increased from 75.8% in phase 3 to 90.7% in phase 4. There was a fall in aggregate defined daily dose to 658.4, from 808.2 per 1000 patient days, and length of therapy also improved. There was a marked increase in the number of culture-based interventions. The educational programs and multidisciplinary team rounds further reinforced the practice of AMS.

**Conclusions:**

The PDCA cycle significantly improved the use of antimicrobials, adherence to treatment guidelines, and patient outcomes in a resource-constrained setting. This approach provides a scalable model for implementing AMS in surgical units despite challenges such as limited microbiological resources.

## Introduction

Since their discovery, antimicrobials have been an important aspect of health care. However, their use is often inappropriate, excessive, or even misaligned with the clinical goals. This irrational antimicrobial use leads to raised healthcare costs, and adverse effects, which are avoidable via implementing systematic programs promoting appropriate antimicrobial prescription.^1–4^

Antimicrobial stewardship (AMS) involves strategies to optimize antimicrobial application, for selection, dosage, and duration of therapy (DOT), with an aim to achieve the optimal clinical outcome while minimizing adverse events.^5,6^ Strategies such as prospective audit and feedback, dose optimization, de-escalation of therapy, and DOT optimization have been proven to reduce metrics such as the number of defined daily doses given, DOT, the proportion of patients on redundant antibiotics (such as double anaerobic or double gram-negative coverage), number of antibiotics prescribed per patient, and length of hospital stay while assisting in obtaining information on prevalent organisms and their sensitivity patterns in tertiary care settings.^7,8^ However, a multidisciplinary strategy is necessary to implement AMS efficiently.^9^

Antimicrobial agents are commonly prescribed for trauma patients either empirically or prophylactically due to the risk of infections. The Advanced Trauma Center (ATC) is a dedicated facility for the management of trauma cases and is an important tertiary care referral center. The patients admitted here are often suffering from multiple traumas and quite often require multiple interventions. Since it is a referral center, they are often referred after initial management at a peripheral center. However, the facility treats any patient who reports at the emergency outpatient unit. It has a multispecialty faculty with expertise in orthopedics, neurosurgery, surgery, and plastic surgery with support from other departments in the main hospital which is also a tertiary care center. In the wake of the high antimicrobial use noted in this facility, it was decided to initiate a plan-do-check-act (PDCA) cycle under the AMS program of the institute.

The PDCA cycle, known as the Deming circle or Shewhart cycle, consists of four stages in a continuous cycle. The PDCA cycle provides a structured and iterative approach to address issues, improve processes, and enhance the overall quality of care and services. The PDCA cycle has proven to be highly effective in hospital quality management, and currently, it is widely employed to standardize practices and improve the quality of patient care.^10–12^

This study aims to reduce excessive and inappropriate antibiotic use in the ATC over four years (March 2019 to May 2023) by applying the PDCA cycle. Additionally, the study seeks to document challenges related to implementing AMSP interventions.

## Methods

### Study setting

The study was conducted at a tertiary government health care and research institute and referral center in North India with over 2000 beds available for inpatients. The hospital sees around 2.5 million patients annually, with super specialty clinics providing care for more than 80,000 patients yearly. Approximately 150 beds, including intensive care units, are available at the hospital’s ATC. The ATC received 25,942 patients in 2022 via new admissions and referrals from other hospitals. Approximately 60% of this load were orthopedic patients. A group of physicians, department faculty, and Junior and senior residents (SRs) provide emergency care around the clock.

### Study period

**Figure 1.**
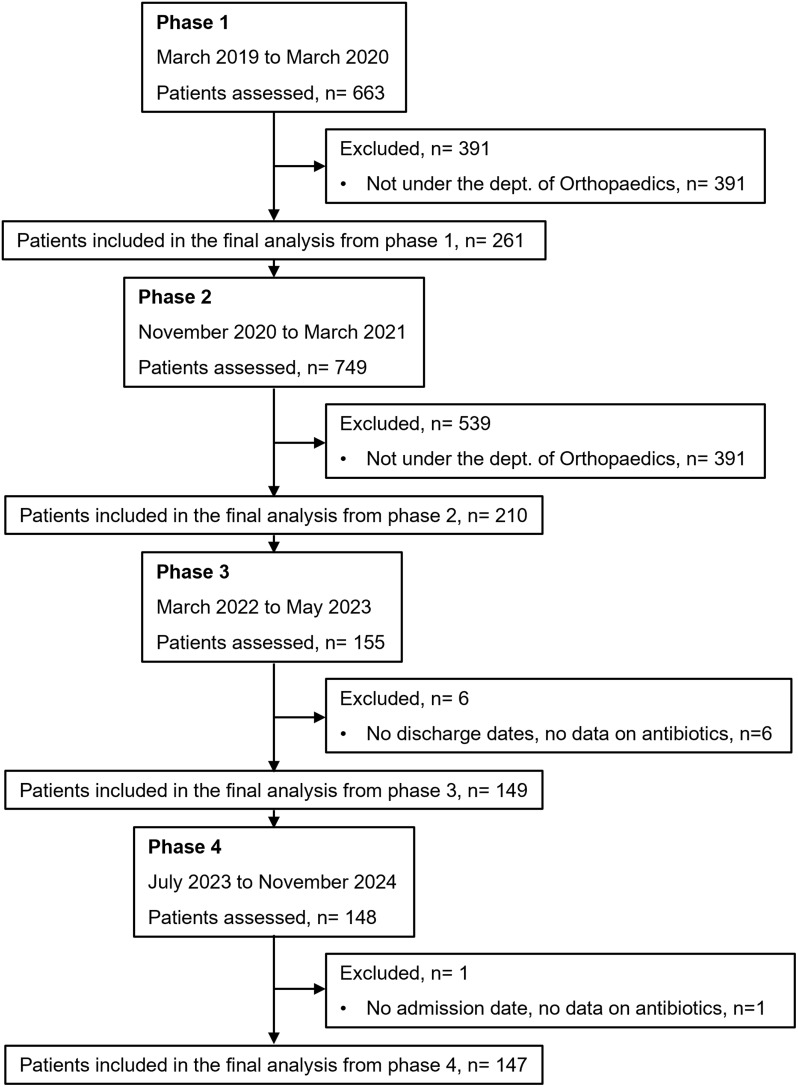
CONSORT flow diagram. The progression of patients through the four phases of the study is depicted. Data of patients admitted in the ATC under departments other than the orthopedics department were also recorded but excluded from the final analysis. Patients with inadequately filled data forms were also excluded from the final analysis.

The study was executed in four phases. Phase 1 was executed from March 2019 to March 2020. Due to the shifted national and institutional commitments toward the containment of the COVID-19 pandemic, phase 2 had a delayed start from November 2020; and ended during March 2021. Phase 3 was conducted from March 2022 to May 2023; and phase 4, from July 2023 to November 2024 (Figure 1).

### Study design

This study was conducted as implementation research, with each phase representing a sequential iteration of the PDCA cycle; no attempt was made to compare phases as equivalent cohorts or to draw causal inferences between phases.

Prior to the program, an expert technical committee was established, including some of the institute’s AMS committee members and faculty members from the Departments of Pharmacology, Orthopaedics, Microbiology, and Hospital Administration.

#### Phase 1

In this phase, the prospective audit was conducted by an AMS nurse, who was in charge of the ATC, and based on this, feedback and suggestions on the antimicrobial prescribing patterns were forwarded to the nursing officers (NOs), SRs, and junior residents (JRs) posted in the ATC wards. Inversely, feedback was also taken from these NOs and residents. Combined meetings were conducted to facilitate this process.

#### Phase 2

Phase 2 focused on cases with fractures, and on educating residents and NOs about proper hand hygiene and sample collection practices. A system for tracking all samples that are sent to the Microbiology laboratory was established. Initiatives for making decisions based on these reports were taken. Additionally, the combined meetings during this phase aim to facilitate resident-to-resident feedback on antimicrobial prescription practices, fostering a culture of continuous improvement and collaboration among the healthcare team.

#### Phase 3

Phases 3 and 4 focused on open fractures. Phase 3 involved the adoption of multi-modal communication to facilitate case discussions and knowledge exchange among faculty and residents. Toward this end, a group was formed, including faculty and residents from the Department of Orthopaedics and Pharmacology. Relevant cases were posted in this group and discussed in brief online, and in further detail during combined rounds. These rounds were held every Friday, involving participation from both departments, as well as from the NOs. These rounds were followed by “bedside bite-size” educational sessions. A total of 24 rounds and 3 bedside bite-size sessions were done.

The residents from the Department of Orthopaedics staffing the ATC were also instructed to post any microbiological case reports of patients with MDR pathogens, as soon as they went online in the HIS (Hospital Information System), should they find the case to be challenging. An updated advice on antimicrobial use was given by the antimicrobial steward for policy related to antibiotics use, and availability and positioning of hand rubs. This phase also included more consultant-to-consultant feedback.

#### Phase 4

Phase 4 included all the above, with greater involvement of NOs and trainee nurses in following infection prevention measures, with emphasis on the earliest removal of any unnecessary cannulas and catheters. There was more involvement of the Infection Control Committee in the PDCA program. There was an enhanced emphasis on laboratory-based antimicrobial therapy.

Across all phases, “Check” activities involved audit-based monitoring of antimicrobial use metrics and compliance with feedback. “Act” activities involved refinement of stewardship strategies informing the design and focus of subsequent phases (Supplementary Table 1).

### Study population

The study covered patients hospitalized for more than 24 hours at the ATC under the Department of Orthopaedics, between March 2019 and November 2024.

### Data collection

Data collected included details regarding patient demographics (age, weight, and sex), admission details, previous hospitalization (whether case is referred or not, previous hospitals admitted, durations of previous hospitalizations, and drugs received previous), injury (data, cause, and nature), procedure (type of surgeries performed and date), antimicrobial prescription (reason, rationality [prophylactic, empiric or definitive] route, dose, and frequency), vitals, and reports (CBC, LFT, RFT, serum electrolytes, radiological investigations, and microbiology). The primary source of patient information was the history given by the patients or their attendants, inpatient case files, and the HIS.

The use of local or topical antibiotics (eg, antibiotic beads or powders) was not routinely practiced, only systemic antibiotic use was methodically captured.

### Study outcomes

These were derived from the primary data.

Duration of stay

Number of patients on antibiotics

Number of antibiotics prescribed per patient

Compliance with standard treatment guidelines (ie, the prophylactic antimicrobial policy of the Orthopaedics Department of PGIMER, Chandigarh)

Defined daily dose (DDD expressed per 1,000 patient days): the total consumption of antibiotics expressed in terms of the DDD, which is the assumed average maintenance dose per day for a drug used for its main indication in adults, according to the World Health Organization (WHO). It is calculated using the formula:

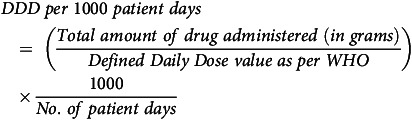




This outcome standardizes antibiotic usage, allowing comparisons across different settings or periods by normalizing drug consumption relative to a standard dose and patient population size.

Days of therapy (DOT expressed per 1,000 patient days): the total number of antibiotic doses administered, expressed as the number of days a patient receives a single antibiotic agent, regardless of the number of doses per day or route of administration. Each antibiotic administered counts as one DOT per day, and concurrent use of multiple antibiotics is counted separately

Length of therapy (LOT expressed per 1,000 patient days): the total number of days during which a patient receives any antibiotic, counting unique days regardless of the number of antibiotics administered concurrently

### Data analysis

The primary data were fed into an MS Excel datasheet, and derived variables were obtained. These also included rationality in the use of antimicrobials (expressed as a percentage of the total antibiotic usage), proportion of culture-based prescriptions, instances of de-escalation, and redundant antibiotic usage (expressed as a percentage of the total).

Descriptive analysis was performed for all the outcome parameters. Quantitative variables were expressed as mean (SD), whereas qualitative variables were expressed as number (percentage).

## Results

### Study cohort

The number of patients who were followed up through various phases ranged from 147 to 261. The median duration of hospital stay per patient was considerably lower in the first two phases compared to the latter half of the study. Most cases in each phase were referred from other healthcare facilities. Only 21.9% of patients in phase 2 had open fractures, while phases 3 and 4 had thrice this. It should be noted that in the event of a patient having both open and closed fractures or multiple open fractures of varying grades according to the Gustilo and Anderson open fracture classification system, they were given the prophylaxis for the highest grade of open fracture present (Table 1).

**Table 1.** Baseline details. Details on patient demographics, previous hospitalizations, and fractures

Parameters	Phase 1	Phase 2	Phase 3	Phase 4
Patients, n	261	210	149	147
Males, n (%)	229 (87.7)	176 (83.8)	125 (83.9)	124 (84.4)
Females, n (%)	32 (12.3)	34 (16.2)	24 (16.1)	23 (15.6)
Age (years), mean (SD)	35.3 (17.6)	35.7 (16.3)	36.2 (15.8)	34.8 (14.9)
Weight (kg), mean (SD)	57.6 (16.1)	56.8 (13.1)	58.1 (13.9)	58.8 (12.1)
Mode of admission				
Referred, n (%)	225 (86.2)	175 (83.3)	142 (95.3)	103 (70.1)
Direct, n (%)	36 (13.8)	35 (16.7)	7 (4.7)	44 (29.9)
Duration of hospital stay (days), median (IQR)	8 (5, 13)	7 (4, 10)	24 (15, 38)	28 (15, 45)
Patients with open fractures, n (%)	115 (44.1)	46 (21.9)	88 (59.1)	97 (67.0)

SD: standard deviation; IQR: interquartile range.

### Trends in antimicrobial prescriptions

Table 2 gives a picture of the antimicrobial prescription trends during the four phases.

**Table 2.** Antimicrobial prescription during the four phases

Parameters	Phase 1 (N = 261)	Phase 2 (N = 210)	Phase 3 (N = 149)	Phase 4 (N = 147)
Patients on antimicrobials, n^*^ (%)	134 (51.3)	94 (44.8)	135 (90.6)	146 (99.3)
Patients on 1 antimicrobial, n^*^ (%)^†^	60 (44.8)	34 (36.2)	35 (25.9)	42 (28.8)
Patients on 2 antimicrobials, n^*^ (%)^†^	20 (14.2)	10 (10.6)	23 (17.0)	46 (31.5)
Patients on ≥3 antimicrobials, n^*^(%)^†^	56 (41.0)	50 (53.2)	77 (57.0)	58 (39.7)
Total number of antimicrobials prescribed, n				
Rationale for prescription:	270	208	402	384
Prophylactic, n^‡^ (%)	250 (92.6)	155 (74.5)	201 (50.0)	172 (44.8)
Empirical, n^‡^ (%)	18 (6.7)	51 (24.5)	122 (30.3)	130 (33.8)
Culture-based, n^‡^ (%)	2 (.7)	2 (1.0)	79 (19.7)	82 (21.4)
Average number of antimicrobials prescribed per patient, mean (SD)	1.03 (1.25)	.99 (1.28)	2.70 (2.02)	2.61 (1.69)

SD: standard deviation.

* Refers to the number of individuals during that phase of the study.

† Expressed as a percentage of the number of patients on antimicrobials in that phase of the study.

‡ Refers to the number of antimicrobials prescribed during that phase of the study.

In the first half of the study, only about half of the patients surveyed (51.3% in phase 1 and 44.8% in phase 2) were on antimicrobials, while 90.6% in phase 3 and almost all the patients in phase 4 were on antimicrobials. In each phase, more than a third of those receiving antimicrobials were on three or more antibiotics.

The team evaluated 270 antimicrobial prescriptions in phase 1 and 208 in phase 2. This number was doubled to 402 in phase 3 and 384 in phase 4. Thus, in the first half of the study, each patient surveyed had an average of approximately 1 antimicrobial prescribed, while this number was tripled in the latter half, with phase 3 patients receiving 2.7 (2.02) antimicrobials on average and phase 4 patients receiving 2.61 (1.29).

**Figure 2.**
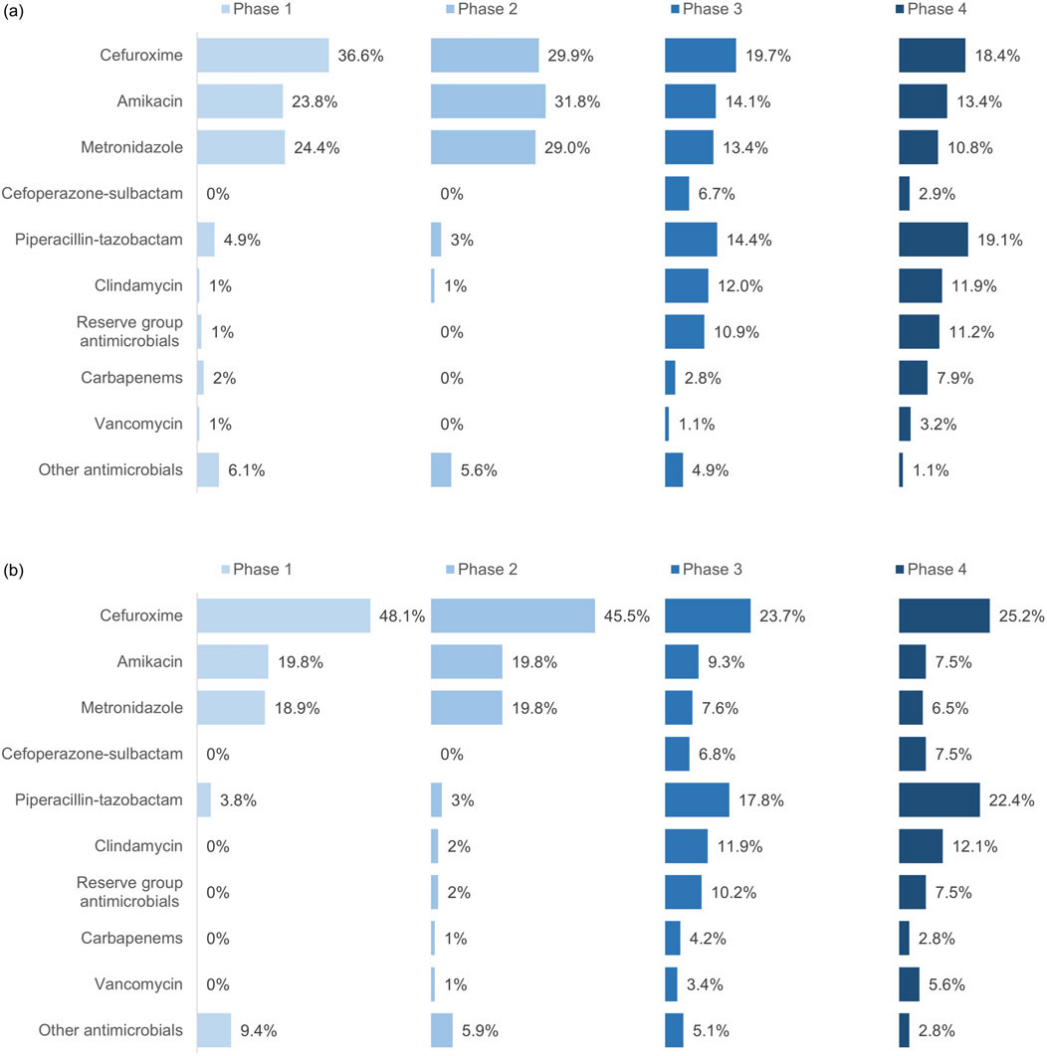
Overall antimicrobial prescription data from each phase. a. Overall antimicrobial prescriptions in patients with open fractures. b. Overall antimicrobial prescriptions in other patients. Values are expressed as a percentage of the total number of individual prescriptions. The reserve group includes aztreonam, colistin, linezolid, minocycline, and tigecycline. Other antimicrobials include penicillin-group antibiotics (amoxicillin, amoxicillin-clavulanic acid, cloxacillin), other cephalosporins (ceftriaxone, ceftazidime, cefepime, cefixime), ciprofloxacin, doxycycline, gentamicin, and teicoplanin.

**Figure 3.**
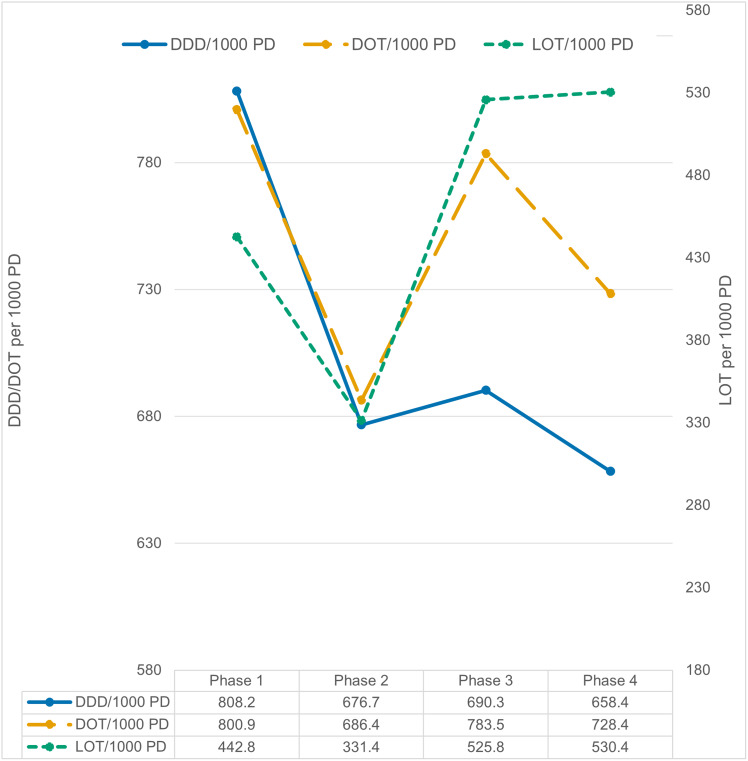
Comparison of DDD, DOT, and LOT values through the four phases. The left y-axis represents the aggregate DDD/1,000 patient days (blue, solid line) and aggregate DOT/1,000 patient days (yellow, dashed line) and is truncated from 580 units. The right y-axis represents LOT/1,000 patient days (green, dotted line) and is truncated from 180 units. A data table is provided at the bottom of the figure.

**Figure 4.**
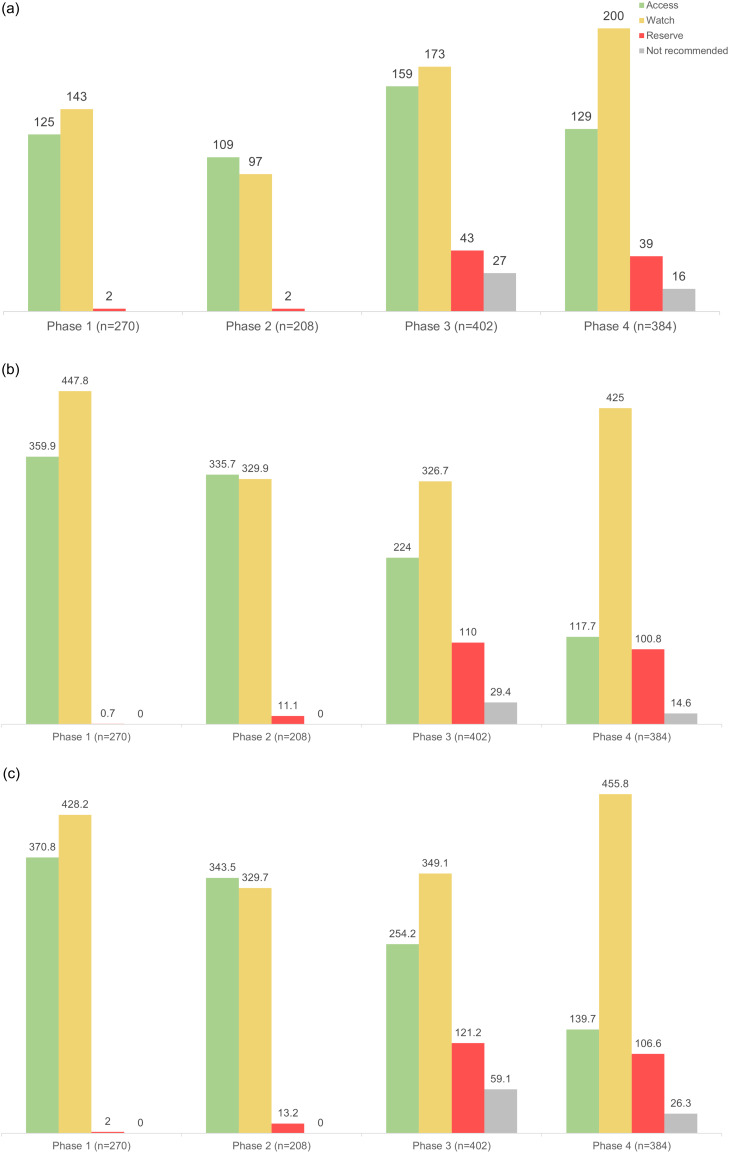
Drug utilization metrics for the AWaRe drug categories (Access, Watch, Reserve, and Not Recommended) across study phases, illustrating the changing trends in antimicrobial usage patterns. (a) Number of drug prescriptions among AWaRe drug categories of antimicrobials. (b) Cumulative DDD values for the AWaRe drug categories. Values are in DDD/1,000 patient days. c. Cumulative DOT values for the AWaRe drug categories. (Values are in DOT/1,000 patient days.).

An overwhelming majority of antimicrobials prescribed in phase 1 were prophylactic (92.6%), with only 6.7% given as empirical. Through the phases, the proportion of empirical prescriptions increased, with phase 2 at 24.5%, phase 3 at 30.3% and phase 4 at 33.8%. In phases 1 and 2, only two prescriptions were based on microbiology culture and sensitivity reports, whereas this number increased to 19.7% of the total number of antimicrobial prescriptions in phase 3 and to 21.4% in phase 4. The trends of antibiotic use in different phases of the cycle can be seen in Figures 2, 3, and 4 and in Supplementary Tables 2 and 3.

**Table 3.** Feedback data. Number of feedback given, their nature, and compliance of the staff of ATC with the suggestions of the PDCA team

Instances	Phase 1	Phase 2	Phase 3	Phase 4
Number of feedback given	167	197	236	364
Nature of feedback				
De-escalation/antibiotic discontinuation	167	197	133	167
Dose modification	NIL	NIL	18	71
Start appropriate antibiotic	NIL	NIL	34	31
Send culture	NIL	NIL	51	94
Compliance				
Suggestions complied with, n (%)^*^	167 (100)	197 (100)	179 (75.8)	330 (90.7)
Proportion of prophylactic prescriptions in compliance with institutional guidelines, %^¥^	24.8%	31.0%	35.3%	48.8%

* Expressed as a percentage of the number of suggestions given in that phase.

¥ Expressed as a percentage of the number of prophylactic antibiotic prescriptions in that phase.

### Quantitative outcome metrics

The aggregate DDD was 808.2 per 1,000 patient days in phase 1, it became 676.7 in phase 2 and was 690.3 and 658.4 in phases 3 and 4, respectively. The aggregate DOT per 1,000 patient days was remaining in the 680 to 800 range. The aggregate length of therapy was 444.2 per 1,000 patient days in phase 1. Later, the aggregate LOT/1,000 PD fell to 331.4 in phase 2 and increased to 525.8 in phase 3 and 530.4 in phase 4 (Figure 4) (Supplementary Tables 3 and 4).

**Table 4.** Microbiological specimen sent during phase 3. Details of specimens sent for microbiological analysis from patients documented during phase 3

Specimen send	Results obtained		
	Sterile/negative	Non-specific^*^	Specific organism(s)
For AFB			
Tissue	2	–	–
ET aspirate	1	–	–
Sputum	2	–	–
For viral serology			
Blood (viral markers)	3	–	–
For bacteriological testing			
Pus	12	12	64
Wound swab	14	21	64
Fluid from drains	–	–	4
Fluid from abscesses	–	–	1
Tissue	4	1	12
Bone	1	1	1
Blood	13	–	3
Urine	3	1	–
ET aspirate	1	2	8

AFB: acid fast Bacilli, ET Aspirate: Endotracheal Aspirate.

* Non-specific details include insignificant bacterial growth, mixed flora with possible contamination necessitating re-send of specimen following proper sampling practices, and gram-negative bacilli or gram-positive cocci detected during Gram stain which were not cultured.

Mirroring the trends in antimicrobial prescriptions, the majority contribution to the aggregate DDD and DOT data during phases 1 and 2 was from the access and watch group of drugs. However, in the last two phases, the contribution from the watch and reserve categories increased much more compared to that from the access group. This was due to the comparatively higher durations of therapy for which these drugs were given (Figure 3).

### Qualitative outcome metrics

Suggestions on antimicrobial prescriptions were given as feedback to the residents and NOs staffing the ATC during all four phases. Thus, the number of feedback given more than doubled from phase 1 (167) through phase 4 (364) (Table 3).

In the first two phases, the only suggestions given were toward either de-escalation of antibiotic use, or stoppage. These were met with 100% compliance. However, in their latter half, suggestions included adjustment of doses, new antibiotic prescriptions and requests to send cultures to guide further course of action. These latter phases saw an initially reduced compliance with our suggestions in phase 3 (75.8%), which rose to 90.7% in phase 4 (Table 3). Nevertheless, this has led to an acute increase in culture-based interventions during the latter phases (Table 2). Furthermore, the proportion of prophylactic prescriptions in compliance with institutional guidelines doubled throughout the study, from 24.8% in phase 1 to 48.8% in phase 4 (Table 3).

### Microorganism profile in culture-confirmed infection cases

**Figure 5.**
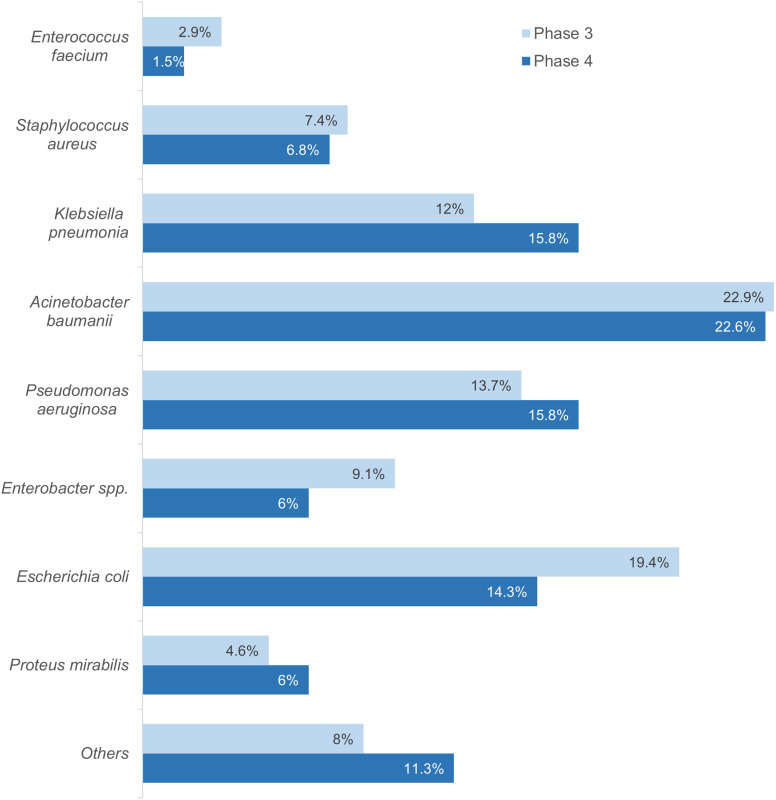
Distribution of bacterial isolates identified in phases 3 and 4 of the study. The chart represents the prevalences of various bacterial species isolated during phases 3 and 4, as a percentage of the total number of isolates cultured. The chief pathogens exhibiting MDR belonging to the ESKAPEE group have been emphasized, along with *Proteus mirabilis* and the remaining bacteria have been grouped under “Others”.

**Figure 6.**
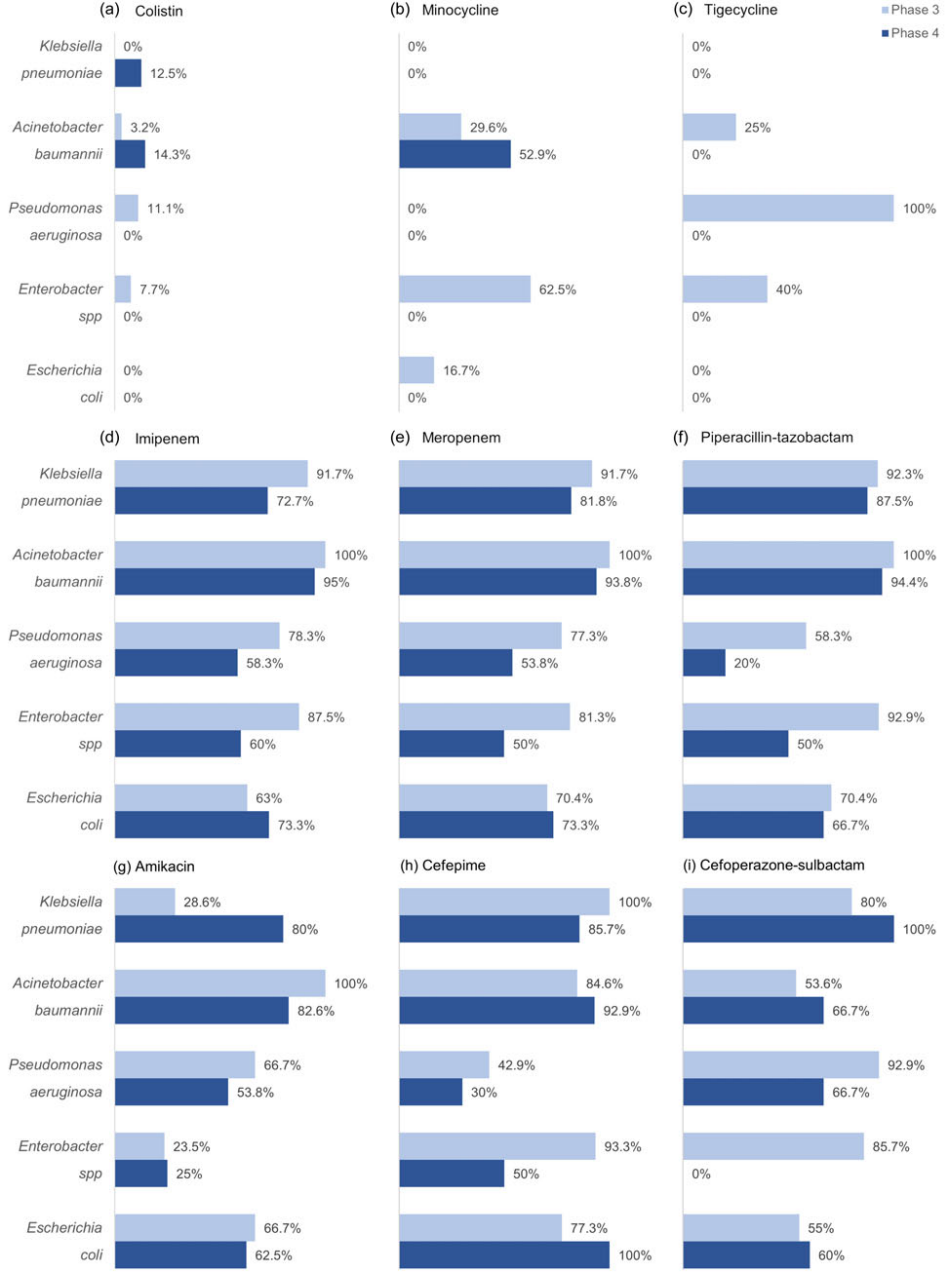
Resistance patterns among gram-negative isolates. The proportion of gram-negative isolates exhibiting resistance to the major antimicrobials used against them. The gram-negative pathogens among the ESKAPEE group are shown here.

The profiles of the specimens sent for microbiological analysis during phases 3 and 4 have been elaborated (Tables 4 and 5). Microbiological analysis during phases 3 and 4 revealed Acinetobacter baumannii as the most common pathogen (22.9% and 22.6%, respectively), followed by Escherichia coli, Pseudomonas aeruginosa, and Klebsiella pneumoniae. Resistance trends showed significant resistance to carbapenems and piperacillin-tazobactam among gram-negative pathogens, while A. baumannii exhibited notable resistance to tigecycline and minocycline. Gram-positive pathogens like Staphylococcus aureus and Enterococcus faecium were sensitive to vancomycin, linezolid, and doxycycline, despite an increase in methicillin-resistant S. aureus (Figure 5, 6) (Supplementary tables 5, 6, 7, and 8).

**Table 5.** Microbiological specimen sent during phase 4. Details of specimens sent for microbiological analysis from patients documented during phase 3

Specimen send	Results obtained		
	Sterile/ negative	Non- specific^*^	Specific organism(s)
For AFB			
Pus	1	–	–
Wound swab	1	–	–
Tissue	1	–	–
ET aspirate	2	–	–
For mycological testing			
Pus^†^	3	–	–
For bacteriological testing			
Pus	20	14	66
Wound swab	4	6	11
Discharge from bone	–	–	1
Fluid from drains	2	–	–
Other serous discharge from wound	–	–	4
Tissue	6	7	13
Bone	1	–	3
Blood	33	1	3
Urine	9	–	–
ET aspirate	9	6	12
Nasal swab	1	–	-

AFB: acid fast bacilli, ET aspirate: endotracheal aspirate.

* Non-specific details include insignificant bacterial growth, mixed flora with possible contamination necessitating re-send of specimen following proper sampling practices, and gram-negative bacilli or gram-positive cocci detected during Gram stain which were not cultured.

† Calcofluor white staining was done toward mycological testing.

## Discussion

We have conducted an extensive study by implementing PDCA on AMS interventions in trauma settings throughout 4 phases in which active feedback was the vital priority. The objectives were to improve patient outcomes, optimize antibiotics, and promote the rational use of antimicrobials.

Antibiotic prophylaxis in orthopedic surgery reduces the risk of surgical site infections. The American Academy of Orthopaedic Surgeons (AAOS) has recommended in its clinical practice guideline (2019) that perioperative antibiotics should be chosen based on each institution’s principles of responsible stewardship.^13^ Appropriate antibiotic use: antibiotic selection, timing, and duration are considered according to institutional antibiotic guidelines. The use of prophylactic antibiotics is a balance between reducing the incidence of SSI whilst maintaining proper antibiotic stewardship to limit adverse effects of the injudicious use of antibiotics.

We have implemented AMS intervention with a low-hanging fruit WHO strategy^14–15^; the initial phases focused on NOs, involving residents and combined rounds with faculty to facilitate the intervention at each phase. Infection prevention and control were given importance during all the phases and have been intensified during phase 4.

We have analyzed four phases till November 2024. Various strategies were formulated across the phases, including dissemination of institutional antibiotic protocol for open and closed fractures, educating trauma nurses and resident doctors through bedside bite-size educative sessions, prospective audit and feedback, creating a group involving orthopedics faculty and residents to post select orthopedic cases for regular ASP rounds, microbiology report tracking and intensification of infection control measures.

A total of 767 patients were engaged in this study which lasted over half a decade. Considerable improvements in various outcome parameters were observed through this program when we observed the results of phase 4 compared to what we noticed during phase 1. The distribution of multidrug-resistant isolates remained relatively consistent between the phases within this study and showed concordance with previous data collected.^16^

In a tertiary care center, referred cases of multiple open fractures with potential risk of infection are a considerable challenge for rational antibiotic use. Importantly, a large proportion of antimicrobial use was driven by infection due to gram-negative pathogens. It is important to understand that due to heavily loaded systems and limited human and infrastructure, delays in follow-up surgeries, infection prevention and control become very challenging. However, it is well understood that the key to decreasing antimicrobial use in the facility is two-fold- reducing the inflow of patients and strengthening infection prevention measures. Steps toward the same are being undertaken and in the ongoing PDCA cycle, efforts toward the same are expected to yield more fruitful results. Importantly, the report would serve as a template for the initiation of such activities in predominantly surgical units to reduce the load of antimicrobial use. We have demonstrated that implementing an efficient ASP with the oversight of an effective PDCA system, repeated over multiple iterations, can drastically improve prescribing practices to improve treatment outcomes.

The “two-step” prospective audit and feedback strategy involving assessment of rationality by available consultant/senior team member implemented here imparts added requirements of expertise, complexity, and time-intensiveness to the program, thus limiting the expansion of the services to further wards and ICUs. The adoption of a standardized algorithmic approach toward the assessment of antimicrobial rationality can be a viable solution to this limitation.^17–20^ A rather inadequate amount of data on cultured isolates was obtained, making it impossible to provide meaningful data on the culture sensitivity patterns of gram-positive isolates or to create an antibiogram.^21^ Albeit these not being the primary objectives of the study, they would have assisted in the formulation of future antimicrobial policies in this setting. Another limitation is the over-emphasis on quantitative measures, providing oversight on patient-centered qualitative metrics for improved education among residents, NOs, and patients. Additionally, heterogeneity in patient characteristics across phases reflects the evolving clinical focus of the PDCA cycle and limits direct phase-to-phase comparison, necessitating interpretation of findings as process-level trends rather than effect estimates.

Despite the challenges and limitations, the analysis of the 4-phase PDCA cycle implemented herewith illuminates the effectiveness of such multidisciplinary interventions in optimizing antimicrobial usage in resource-limited tertiary healthcare settings.

## Supporting information

10.1017/ash.2026.10374.sm001 P et al. supplementary material P et al. supplementary material

## Supplementary material

The supplementary material for this article can be found at https://doi.org/10.1017/ash.2026.10374.

## Further information

### Antibiotic resistance

The guidelines on prevention of Surgical Site Infections issued by the US Centers for Disease Control and Prevention (CDC) (2019) recommended that only a single dose of Surgical Antimicrobial Prophylaxis is required peri- operatively for clean-contaminated procedures, including arthroplasty procedures. This was based on evidence and findings in orthopedic surgery and other surgical specialties.

### Empirical antibiotic treatment

Initial antibiotic treatment targeting the most probable causative microorganism. The recommendations should be based on local susceptibility data and available scientific evidence or expert opinion when evidence is lacking.

### Structural measures/indicators for AMS programs

Structure refers to the characteristics (capacity, systems, and processes) of the setting in which AMS programs are conducted. Structures may be material or human resources, such as availability of financial resources, number of personnel, availability of guidelines, availability of information technology tools, etc.
