# Supplementary material for: Plan-do-check-act (PDCA) cycle analysis for antimicrobial stewardship of orthopedic patients in trauma center—implementation research
Source: Antimicrob Steward Healthc Epidemiol. 2026 May 11;6(1):e126. doi: 10.1017/ash.2026.10374 (PMC13162072; doi:10.1017/ash.2026.10374)
Supplement: P et al. supplementary material [file S2732494X2610374Xsup001.docx]

| **PDCA Aspect** | **Phase 1** **(Mar 2019-Mar 2020)** | **Phase 2** **(Nov 2020-Mar 2021)** | **Phase 3** **(Mar 2022-May 2023)** | **Phase** 4 **(Jul 2023-Nov 2024)** |
| --- | --- | --- | --- | --- |
| **PLAN** | Identification of high prophylactic and empirical antimicrobial use; lack of structured stewardship oversight in ATC; formation of multidisciplinary AMS–PDCA team | Identification of gaps in hand hygiene, sample collection, and microbiology utilization in fracture patients | Identification of stewardship challenges in open fractures and MDR infections; need for rapid interdisciplinary communication | Identification of persistent infection-prevention gaps and delayed laboratory-driven decision-making |
| **DO** | Prospective audit of antimicrobial prescriptions; feedback to residents and nursing officers; combined meetings | Education on hand hygiene and sample collection; tracking of microbiology samples; resident-to-resident feedback | Multimodal communication via interdisciplinary group; weekly combined rounds; bedside “bite-size” education; real-time microbiology consultation | Intensified infection prevention measures; increased nursing involvement; emphasis on early removal of invasive devices; enhanced lab-based therapy |
| **CHECK** | Review of antimicrobial prescription patterns; monitoring of feedback acceptance | Monitoring of sample submission rates and early microbiology reporting; review of prescribing practices | Audit of DDD, DOT, LOT; monitoring compliance with stewardship feedback; assessment of culture-based prescribing | Continued audit of utilization metrics and compliance; review of microbiological trends and prescribing appropriateness |
| **ACT** | Initiation of structured stewardship feedback and education | Refinement of stewardship focus toward fracture-related infections and diagnostic stewardship | Expansion of stewardship activities to complex open fractures; escalation of consultant-to-consultant feedback | Strengthening of laboratory-supported therapy and infection control integration; consolidation of stewardship practices |

**Supplementary Table 1. PDCA Phases.** Phase-wise implementation of the Plan–Do–Check–Act (PDCA) cycle for antimicrobial stewardship in the orthopaedic trauma unit

|  | **P1O** | **P1C** | **P2O** | **P2C** | **P3O** | **P3C** | **P4O** | **P4C** |
| --- | --- | --- | --- | --- | --- | --- | --- | --- |
| Cefuroxime | 49 | 51 | 32 | 46 | 57 | 28 | 51 | 27 |
| Amikacin | 39 | 22 | 34 | 20 | 40 | 11 | 37 | 8 |
| Metronidazole | 42 | 21 | 31 | 20 | 38 | 10 | 30 | 7 |
| Piperacillin-tazobactam | 9 | 4 | 3 | 3 | 43 | 21 | 53 | 24 |
| Clindamycin | 1 | - | 1 | 2 | 36 | 14 | 33 | 13 |
| Colistin | 2 | - | - | 2 | 16 | 9 | 19 | 5 |
| Cefoperazone-sulbactam | - | 1 | - | - | 19 | 9 | 8 | 8 |
| Vancomycin | 3 | - | - | 1 | 7 | 4 | 15 | 6 |
| Cefixime | 12 | 3 | - | - | 1 | - | 1 | - |
| Ceftriaxone | 6 | 10 | 6 | 5 | 1 | 2 | 0 | 1 |
| Meropenem | 1 | - | - | 1 | 3 | 5 | 9 | 3 |
| Tigecycline | - | - | - | - | 9 | 2 | 2 | - |
| Amoxicillin-Clavulanic acid | 4 | 1 | - | 1 | 5 | 1 | - | - |
| Minocycline | - | - | - | - | 3 | 2 | 6 | 1 |
| Imipenem | - | - | - | - | 1 | - | 7 | - |
| Linezolid | - | - | - | - | 3 | - | 3 | 2 |
| Doxycycline | - | - | - | - | 3 | - | 1 | - |
| Ciprofloxacin | - | 1 | - | - | - | 1 | 1 | - |
| Cloxacillin | - | - | - | - | 1 | 2 | - | - |
| Ceftazidime | - | - | - | - | 1 | - | - | 1 |
| Amoxicillin | - | - | - | - | 1 | - | - | - |
| Aztreonam | - | - | - | - | - | - | 1 | - |
| Cefepime | - | - | - | - | 1 | - | - | - |
| Gentamicin | - | - | - | - | 1 | - | - | - |
| Teicoplanin | - | - | - | - | - | - | - | 1 |

**Supplementary table 2. Antimicrobial prescription frequencies patterns across four phases of the study.** Data represent the frequency of prescriptions for each drug, among patients with open fractures and among those without, across the four phases of the study.
P1O: Phase 1, Patients with open fractures; P1C: Phase 1, Patients without open fractures;
P2O: Phase 2, Patients with open fractures; P2C: Phase 2, Patients without open fractures;
P3O: Phase 3, Patients with open fractures; P3C: Phase 3, Patients without open fractures;
P4O: Phase 4, Patients with open fractures; P4C: Phase 4, Patients without open fractures

| **Antimicrobial** | **Phase 1** | **Phase 2** | **Phase 3** | **Phase 4** |
| --- | --- | --- | --- | --- |
| Amikacin | 188.0 | 174.6 | 55.5 | 38.9 |
| Amoxiclav | 8.9 | 1.7 | 9.6 | - |
| Clindamycin | 4.7 | 4.0 | 61.3 | 37.1 |
| Metronidazole | 158.3 | 155.4 | 62.9 | 36.3 |
| Amoxicillin | - | - | 1.2 | - |
| Cloxacillin | - | - | 14.9 | - |
| Doxycycline | - | - | 17.2 | 5.4 |
| Gentamicin | - | - | 1.4 | - |
| Ceftriaxone | 67.6 | 45.5 | 15.2 | 0.6 |
| Cefuroxime | 326.1 | 248.7 | 140.5 | 159.3 |
| Ciprofloxacin | 2.8 | - | 1.2 | 0.8 |
| Meropenem | 3.2 | 6.5 | 16.7 | 22.7 |
| Piperacillin-tazobactam | 44.9 | 24.3 | 125.8 | 167.7 |
| Vancomycin | 3.2 | 4.9 | 17.8 | 46.4 |
| Cefepime | - | - | 0.5 | - |
| Cefixime | - | - | 5.3 | 0.8 |
| Ceftazidime | - | - | 0.7 | 1.7 |
| Imipenem | - | - | 3.0 | 22.7 |
| Teicoplanin | - | - | - | 2.3 |
| Colistin | 0.7 | 11.1 | 62.3 | 61.9 |
| Linezolid | - | - | 8.6 | 12.6 |
| Minocycline | - | - | 9.8 | 19.2 |
| Tigecycline | - | - | 29.3 | 6.3 |
| Aztreonam | - | - | - | 0.8 |
| Cefoperazone-sulbactam | - | - | 29.4 | 14.6 |

**Supplementary table 3. Cumulative DDD per 1000 patient days for specific antibiotics during each phase of the study.**

| **Antimicrobial** | **Phase 1** | **Phase 2** | **Phase 3** | **Phase 4** |
| --- | --- | --- | --- | --- |
| Amikacin | 182.4 | 174.6 | 52.3 | 36.0 |
| Amoxiclav | 12.3 | 1.7 | 11.9 | - |
| Clindamycin | 7.1 | 6.9 | 90.0 | 59.6 |
| Metronidazole | 169.0 | 160.3 | 73.0 | 41.2 |
| Amoxicillin | - | - | 4.0 | - |
| Cloxacillin | - | - | 10.9 | - |
| Doxycycline | - | - | 9.3 | 2.9 |
| Gentamicin | - | - | 2.8 | - |
| Ceftriaxone | 45.1 | 32.2 | 11.4 | 0.6 |
| Cefuroxime | 327.3 | 247.6 | 141.9 | 147.8 |
| Ciprofloxacin | 2.8 | - | 1.2 | 0.8 |
| Meropenem | 4.7 | 9.8 | 14.9 | 27.4 |
| Piperacillin-tazobactam | 45.5 | 30.4 | 149.3 | 201.3 |
| Vancomycin | 2.8 | 9.8 | 20.2 | 51.4 |
| Cefepime | - | - | 0.9 | - |
| Cefixime | - | - | 5.3 | 0.8 |
| Ceftazidime | - | - | 0.9 | 3.3 |
| Imipenem | - | - | 3.0 | 19.7 |
| Teicoplanin | - | - | - | 2.5 |
| Colistin | 2.0 | 13.2 | 67.9 | 65.2 |
| Linezolid | - | - | 8.6 | 12.3 |
| Minocycline | - | - | 12.3 | 20.7 |
| Tigecycline | - | - | 32.3 | 6.7 |
| Aztreonam | - | - | - | 1.7 |
| Cefoperazone-sulbactam | - | - | 59.1 | 26.3 |

**Supplementary table 4. Cumulative DOT per 1000 patient days for specific antibiotics during each phase of the study.**

| **Bacteria** | **Phase 3** | **Phase 4** |
| --- | --- | --- |
| *Acinetobacter baumannii* | 40 | 30 |
| *Escherichia coli* | 34 | 19 |
| *Pseudomonas aeruginosa* | 24 | 21 |
| *Klebsiella pneumoniae* | 21 | 21 |
| *Staphylococcus aureus* | 13 | 9 |
| *Enterobacter cloacae* | 10 | 4 |
| *Proteus mirabilis* | 8 | 8 |
| *Enterobacter hormaechei* | 6 | 3 |
| *Enterococcus faecium* | 5 | 2 |
| *Pseudomonas mendocina* | 3 | 1 |
| *Citrobacter koseri* | 2 | 1 |
| *Providencia stuartii* | 2 | 1 |
| *Achromobacter xylosoxidans* | 1 | - |
| *Citrobacter freundii* | 1 | - |
| *Clostridium perfringens* | 1 | - |
| *Pseudomonas stutzeri* | 1 | - |
| *Staphylococcus epidermidis* | 1 | - |
| *Staphylococcus haemolyticus* | 1 | - |
| *Stenotrophomonas maltophilia* | 1 | - |
| *Serratia marcescens* | - | 6 |
| *Aeromonas caviae* | - | 1 |
| *Citrobacter braakii* | - | 1 |
| *Staphylococcus hominis* | - | 1 |
| *Citrobacter sedlakii* | - | 1 |
| *Enterobacter asburiae* | - | 1 |
| *Exiguobacterium aurantiacum* | - | 1 |
| *Morganella morganii* | - | 1 |

**Supplementary table 5. Distribution of bacterial isolates cultured during Phases 3 and 4 of the study.** The table shows the prevalence of various bacterial species isolated during phases 3 and 4 as counts.

| **Microorganism** | **Colistin** | | | **Minocycline** | | | **Tigecycline** | | |
| --- | --- | --- | --- | --- | --- | --- | --- | --- | --- |
|  | **R** | **IS** | **S** | **R** | **IS** | **S** | **R** | **IS** | **S** |
| *Enterococcus faecium* | - | - | - | - | - | 1 | - | - | - |
| *Klebsiella pneumoniae* | - | 7 | 3 | - | - | 2 | - | 4 | 8 |
| *Acinetobacter baumannii* | 1 | 26 | 4 | 8 | 4 | 15 | 1 | - | 3 |
| *Pseudomonas aeruginosa* | 1 | 6 | 2 | - | - | - | 1 | - | - |
| *Enterobacter spp.* | 1 | 12 | - | 5 | 1 | 2 | 4 | 3 | 3 |
| *Escherichia coli* | - | 21 | 3 | 1 | 2 | 3 | - | 3 | 18 |
|  | **Imipenem** | | | **Meropenem** | | | **Piperacillin-tazobactam** | | |
|  | **R** | **IS** | **S** | **R** | **IS** | **S** | **R** | **IS** | **S** |
| *Enterococcus faecium* | - | - | - | - | - | - | - | - | - |
| *Klebsiella pneumoniae* | 11 | - | 1 | 11 | - | 1 | 12 | - | 1 |
| *Acinetobacter baumannii* | 23 | - | - | 22 | - | - | 29 | - | - |
| *Pseudomonas aeruginosa* | 18 | 1 | 4 | 17 | 2 | 3 | 7 | 4 | 1 |
| *Enterobacter spp.* | 14 | - | 2 | 13 | 1 | 2 | 13 | 1 | - |
| *Escherichia coli* | 17 | - | 10 | 19 | - | 8 | 19 | 1 | 7 |
|  | **Amikacin** | | | **Cefepime** | | | **Cefoperazone-sulbactam** | | |
|  | **R** | **IS** | **S** | **R** | **IS** | **S** | **R** | **IS** | **S** |
| *Enterococcus faecium* | - | - | - | - | - | - | - | - | - |
| *Klebsiella pneumoniae* | 4 | 9 | 1 | 12 | - | - | 8 | 1 | 1 |
| *Acinetobacter baumannii* | 29 | - | - | 22 | 4 | - | 15 | 6 | 7 |
| *Pseudomonas aeruginosa* | 14 | 2 | 5 | 6 | 4 | 4 | 13 | - | 1 |
| *Enterobacter spp.* | 4 | 10 | 3 | 14 | 1 | - | 12 | 1 | 1 |
| *Escherichia coli* | 18 | 1 | 8 | 17 | 2 | 3 | 11 | 3 | 6 |

**Supplementary table 6. Antimicrobial susceptibility testing results for isolates of the major gram negative pathogens with a higher propensity to exhibit multidrug resistance, cultured during phase 3.** The gram negative members of the ESKAPEE group of organisms are shown here, and their susceptibility towards nine antimicrobials are tabulated. Susceptibility cut-offs were as per Clinical and Laboratory Standards Institute (CLSI) guidelines. R: Resistant IS: Intermediately sensitive S: Sensitive

| **Microorganism** | **Colistin** | | | **Minocycline** | | | **Tigecycline** | | |
| --- | --- | --- | --- | --- | --- | --- | --- | --- | --- |
|  | **R** | **IS** | **S** | **R** | **IS** | **S** | **R** | **IS** | **S** |
| *Enterococcus faecium* |  |  |  |  |  |  |  |  |  |
| *Klebsiella pneumoniae* | 2 | 10 | 4 |  |  | 5 |  |  | 1 |
| *Acinetobacter baumannii* | 4 | 18 | 6 | 9 | 2 | 6 |  |  |  |
| *Pseudomonas aeruginosa* |  | 4 | 5 |  |  |  |  |  |  |
| *Enterobacter spp.* | 0 | 3 | 0 | 0 | 1 | 2 | 0 | 0 | 0 |
| *Escherichia coli* |  | 8 | 5 |  | 1 | 1 |  | 1 | 4 |
|  | **Imipenem** | | | **Meropenem** | | | **Piperacillin-tazobactam** | | |
|  | **R** | **IS** | **S** | **R** | **IS** | **S** | **R** | **IS** | **S** |
| *Enterococcus faecium* |  | 1 |  |  |  |  |  |  |  |
| *Klebsiella pneumoniae* | 8 |  | 3 | 9 |  | 2 | 14 |  | 2 |
| *Acinetobacter baumannii* | 19 |  | 1 | 15 |  | 1 | 17 |  | 1 |
| *Pseudomonas aeruginosa* | 7 |  | 5 | 7 |  | 6 | 2 |  | 8 |
| *Enterobacter spp.* | 3 | 0 | 2 | 1 | 0 | 1 | 2 | 1 | 1 |
| *Escherichia coli* | 11 |  | 4 | 11 |  | 4 | 8 |  | 4 |
|  | **Amikacin** | | | **Cefepime** | | | **Cefoperazone-sulbactam** | | |
|  | **R** | **IS** | **S** | **R** | **IS** | **S** | **R** | **IS** | **S** |
| *Enterococcus faecium* |  |  |  |  |  |  |  |  |  |
| *Klebsiella pneumoniae* | 8 |  | 2 | 6 | 1 |  | 6 |  |  |
| *Acinetobacter baumannii* | 19 | 1 | 3 | 13 | 1 |  | 6 | 1 | 2 |
| *Pseudomonas aeruginosa* | 7 |  | 6 | 3 | 2 | 5 | 6 | 1 | 2 |
| *Enterobacter spp.* | 1 | 0 | 3 | 1 | 1 | 0 | 0 | 0 | 2 |
| *Escherichia coli* | 10 |  | 6 | 7 |  |  | 6 |  | 4 |

**Supplementary table 7. Antimicrobial susceptibility testing results for isolates of the major gram negative pathogens with a higher propensity to exhibit multidrug resistance, cultured during phase 4.** The gram negative members of the ESKAPEE group of organisms are shown here, and their susceptibility towards nine antimicrobials are tabulated. Susceptibility cut-offs were as per Clinical and Laboratory Standards Institute (CLSI) guidelines. R: Resistant IS: Intermediately sensitive S: Sensitive

|  | **Phase 3** | | | **Phase 4** | | |
| --- | --- | --- | --- | --- | --- | --- |
|  | **R** | **IS** | **S** | **R** | **IS** | **S** |
| *Staphylococcus aureus* | | | | | | |
| **Oxacillin** | 6 | - | 6 | 8 | - | 1 |
| **Vancomycin** | - | - | 11 | - | - | 6 |
| **Linezolid** | - | - | 6 | - | - | 6 |
| **Teicoplanin** | 1 | - | 8 | - | - | 4 |
| **Doxycycline** | - | - | 8 | - | - | 6 |
| **Clindamycin** | 2 | - | 7 | 3 | - | 5 |
| *Enterococcus faecium* | | | | | | |
| **Vancomycin** | - | - | 4 | - | 1 | 1 |
| **Linezolid** | - | - | 1 | - | 1 | - |
| **Teicoplanin** | - | - | 4 | - | 1 | 1 |

**Supplementary table 8. Antimicrobial susceptibility testing results for isolates of the major gram positive pathogens with a higher propensity to exhibit multidrug resistance, cultured during phases 3 and 4.** Test results for Staphylococcus aureus and Enterococcus faecium are shown here. Susceptibility cut-offs were as per Clinical and Laboratory Standards Institute (CLSI) guidelines. R: Resistant IS: Intermediately sensitive S: Sensitive
